# Comparison on Functional Assays for Gq-Coupled GPCRs by Measuring Inositol Monophospate-1 and Intracellular Calcium in 1536-Well Plate Format

**DOI:** 10.2174/1875397300801010070

**Published:** 2008-07-11

**Authors:** Ke Liu, Steve Titus, Noel Southall, Pingjun Zhu, James Inglese, Christopher P Austin, Wei Zheng

**Affiliations:** NIH Chemical Genomics Center, National Human Genome Research Institute, National Institutes of Health, Bethesda, MD 20892-3370, USA

## Abstract

Cell-based functional assays used for compound screening and lead optimization play an important role in drug discovery for G-protein coupled receptors (GPCRs). Cell-based assays can define the role of a compound as an agonist, antagonist or inverse agonist and can provide detailed information about the potency and efficacy of a compound. In addition, cell-based screens can be used to identify allosteric modulators that interact with sites other than the binding site of the endogenous ligand. Intracellular calcium assays which use a fluorescent calcium binding dye (such as Fluo-3, Fluo-4 or Fura-2) have been used in compound screening campaigns to measure the activity of Gq-coupled GPCRs. However, such screening methodologies require a special instrumentation to record the rapid change in intracellular free calcium concentration over time. The radioactive inositol 1,4,5- triphosphate (IP_3_) assay measures ^3^H-inositol incorporation and is another traditional assay for the assessment of Gq-coupled GPCR activity, but it is not suitable for screening of large size compound collections because it requires a cell wash step and generates radioactive waste. To avoid these limitations, we have optimized and miniaturized a TR-FRET based IP-One assay that measures inositol monophosphate in a 1536-well plate format. This assay is homogenous, non-radioactive and does not require a kinetic readout. It has been tested with the cell lines expressing M_1_ acetylcholine, FFAR1, vasopressin V1b, or Neuropeptide S receptors. The activities of antagonists determined in the IP-One assay correlated well with these measured in the intracellular calcium assay while the correlation of agonist activities might vary from cell line to cell line. This IP-One assay offers an alternative method for high throughput screening of Gq-coupled GPCRs without using costly kinetic plate readers.

## INTRODUCTION

Guanine nucleotide triphosphate binding protein (G protein)-coupled receptors (GPCRs) are the largest and most important family of cell surface receptors for drug development. It is estimated that over 30% of all marketed small-molecule drugs target GPCRs [1]. When binding to appropriate ligands, GPCRs transduce these extracellular stimuli into intracellular second messengers through activation of one or several G proteins including the subtypes of Gs, Gi, and Gq. The Gs subtype activates adenylyl cyclase (AC), and thus increases the intracellular adenosine 3’,5’-cyclic monophosphate (cAMP), a secondary messenger that signals through the activation of protein kinase A (PKA). The Gi subtype inhibits AC and suppresses the signaling in the cAMP/PKA pathway. The Gq subtype activates phosholipase C (PLC), which increases the second messengers inositol 1,4,5-trisphosphate (IP_3_) and diacyl glycerol (DAG), resulting in an increase of intracellular free Ca^2+^ and activation of protein kinase C (PKC) [2].

A variety of methods have been used to screen for compounds that modulate GPCR signaling. These include measuring direct ligand-receptor binding, level of second messengers, receptor internalization, or expression of reporter genes [3,4]. Ligand binding assays are used to determine the binding affinity of compounds to a particular GPCR, either directly (saturation binding) or indirectly (displacement binding). However, this type of assay can not differentiate the function of compounds and is limited by the availability of labeled ligand. Moreover, compounds that bind to a different site on the receptor may not affect the binding of labeled ligand in this type of assay. Reporter gene assays couple GPCR signaling transduction pathway to the expression level of an exogenous enzyme such as luciferase or beta-lactamase. This type of assay is usually very sensitive to stimulation by an agonist and can be used as a functional assay for compound screening. However, the false positive rate in reporter gene assays is often much higher than other types of assays, which is resulted from the compound’s actions on other proteins in the signaling pathway as well as on the receptor protein’s transcription and synthesis. Second messenger assays are usually cell-based and can be used to screen for agonists, antagonists and inverse agonists. Because second messengers are closer to the initial GPCR signaling events, second messenger assays directly reflect the effect of a compound and thus are more trustworthy than the reporter gene assay. Several types of cAMP assays are currently available for screening of compound libraries against the Gs/Gi-coupled receptors [3,4], including fluorescence polarization (FP) [5] , time-resolved fluorescence resonance energy transfer (TR-FRET) such as HTRF (homogeneous time resolved fluorescence) [6] and Lance, AlphaScreen [7], enzyme fragmentation complementation (EFC) 8 and cyclic nucleotide gated ion channel (CNG)-coupled [9] assays.

The radioactive IP_3_ assay has been used to measure the function of Gq coupled GPCRs for many years since the discovery of IP_3_ as a second messenger [10], but the screening throughput of an IP_3_ assay, based on ^3^H-inositol incorporation and ion exchange chromatography, is very small and is not suitable for HTS. Despite the recent improvement of the IP_3_ assay throughput with the scintillation proximity assay (SPA) technology, it still requires a cell wash step and is not practical for compound screening with large compound libraries [11,12]. Alternatively, the function of Gq-coupled GPCRs can be determined by the measurement of intracellular calcium concentration [12,13]. In the last 10 years, the intracellular calcium assays, based on fluorescence dyes including Fluo-3, Fluo-4 or Fura-2 that become fluorescent upon their binding to free Ca^2+^, have been developed and applied for compound screening; along with the available instruments including Fluorescent-Imaging Plate Reader (FLIPR) and Functional Drug Screening System (FDSS). But use of this assay format has been limited by the need for these special and costly florescence kinetic plate readers. Recently, an IP-One assay which utilizes the HTRF detection format has emerged as an alternative method for the functional measurement of Gq-coupled GPCRs [14]. This assay applies a specific antibody to quantify the amount of inositol-1-phosphate (IP_1_) accumulation in cells and it is sensitive since HTRF is used as the detection method. We report here the miniaturization and optimization of this IP-One assay with four GPCRs as well as a comparison of antagonist screens against M_1_ muscarinic acetylcholine receptor expressed in Chinese hamster ovary (CHO) cells using both the IP-One assay and the intracellular calcium assay.

## MATERIALS AND METHODS

### Materials

All cell culture reagents were obtained from Invitrogen (Carlsbad, CA). Carbachol, pirenzepine, atropine and probenecid were purchased from Sigma Aldrich (St. Louis, MO). The 1536-well tissue culture-treated, clear-bottom black plates and solid white plates were purchased from Kalypsys (San Diego, CA). The IP-One HTRF assay kit was obtained from Cisbio (Bedford, MA). The Fluo-4 containing no-wash PBX calcium assay kit was purchased from BD Biosciences (Rockville, MD). A CHO cell line expressing the murine M_1_ muscarinic acetylcholine receptor (CHO-M_1_) was obtained from American Type Culture Collection (ATCC, Manassas, VA)

### Compound Library

A compound library containing 1208 compounds (Library of Pharmacologically Active Compounds, LOPAC) was purchased from Sigma-Aldrich and dissolved in DMSO to a concentration of 10 mM. All compounds in the LOPAC Library were serially diluted in DMSO to fifteen concentrations in 384-well plates at a ratio of the square root of 5 (1:2.236) as described previously [15]. Four sets of the interplate dilution plates were subsequently reformatted into one set of 1536-well plates at 7 μl/well with concentrations ranging from 0.29 μM to 10 mM as the compound source plates.

### Cell Culture and Frozen Cell Preparation

CHO-M_1_ cells were maintained in F12 Kaighn’s media supplemented with 10 % FBS, 100 units/ml penicillin, 100 μg/ml streptomycin and 250 μg/ml geneticin at 37 ºC, 5 % CO_2 _in a humidified atmosphere. For the frozen cell preparation, 5 × 10^6^ cells in 60 ml of media were seeded in each Nunclon triple layer flask (Nalge Nunc International, Rochester, NY) and were cultured for 3 to 4 days to reach 90-95 % confluence. The cells were then detached by incubation with 15 ml 0.12 % trypsin at 37 °C for 3 minutes and centrifuged at 1000 RPM to remove the trypsin solution. The resulting cell pellet was resuspended in the cell freezing media containing 10 % DMSO (Invitrogen) at a density of 4 × 10^7^ cells/ml. One triple layer flask (500 cm^2^) yielded approximately 4 to 5 × 10^7^ cells. Aliquots of cells with 0.5 – 1 ml per vial were put in a Cryo 1°C freezing container (Nalgene Nunc, Rochster, NY) and slowly frozen down overnight in a -80°C freezer at 1°C/min. The frozen cells were then transferred to liquid nitrogen for storage for up to two years.

### Instruments for Liquid Handling and Plate Detection

Cells and other reagents were dispensed into 1536-well plates by either a flying reagent dispenser (FRD) (Aurora Discovery, San Diego, CA) or a Multidrop Combi dispenser (Thermo Fisher Scientific Inc., Waltham, MA). The control compounds were serially diluted in DMSO in 384-well plates manually and then reformatted into 1536-well plates at 7 μl/well using Cybi-well dispensing station (Cybio, Inc., Woburn, MA). A pintool station (Kalypsys, San Diego, CA) was used to transfer 23 nl of compound in DMSO solution to the 1536-well assay plate in which the final DMSO concentration was under 0.5%. A CCD-based imaging plate reader, ViewLux (PerkinElmer, Boston, MA), was used for the detection of IP-One assay plates in the TR-FRET mode with the excitation of 320 nm and the dual emissions of 620 and 665 nm. A kinetic fluorescence plate reader, Functional Drug Screening System (FDSS) 7000 (Hamamatsu Corp., Hamamatsu City, Japan), was used to measure changes in the intracellular free calcium.

### HTRF IP-One Assay

Frozen CHO-M_1_ Cells were resuspended in the culture media and seeded at 4 μl/well (2000 cells/well) in white, solid bottom, tissue culture-treated 1536-well plates. The cells were cultured at 37 ºC, 5 % CO_2_ overnight to allow recovery from the frozen state. Serial dilutions of compounds were then added at 23 nl/well on the second day and incubated for 10 min. The agonist carbachol was prepared in the stimulation buffer (10mM Hepes, 1mM CaCl_2_, 0.5 mM MgCl_2_, 4.2 mM KCl, 146 mM NaCl, 5.5 mM glucose 250mM LiCl 50mM pH7.4) and was added to the assay plate at 1 μl/well. After 30 min incubation with carbachol at 37 ºC with 5 % CO_2_, all wells in the assay plate were dispensed with 1 μl/well of d2-conjugated IP_1_ (d2-IP_1_) and 1 μl/well of Eu^+^ cryptate-conjugated anti-IP_1_ antibody (Eu^+^-Ab), both from the HTRF IP-One kit and prepared in the cell lysis buffer supplied in the kit. After 10 min incubation at room temperature, the assay plates were measured in the TR-FRET detection mode with a ViewLux plate reader. The results were calculated as a ratio of the acceptor fluorescence intensity divided by the donor fluorescence intensity [16].

### Intracellular Calcium Assay

The resuspended frozen cells were plated at 3 μl/well with 2000 cells in black, tissue culture treated, clear bottom 1536-well plates. After overnight incubation at 37 ºC, 5% CO_2_, 2.5 μl of the calcium dye (1x calcium indicator in HBSS with 2.5 mM Probenecid as per manufacturer’s instructions) was dispensed to all wells and plates were incubated at 37 ºC, 5 % CO_2_ for 1 hour followed by 10 min incubation with 23 nl compound in DMSO solution. The assay plates were then placed in the kinetic fluorescence plate reader (FDSS-7000) for a kinetic measurement of changes in intracellular free calcium. The basal fluorescence signal was recorded 6 times at 1 Hz followed by an addition of 1 μl of Carbachol and 4-minute continuously recording at 1 Hz.

### Data Analysis

The primary screening data was first analyzed using software developed internally in NIH Chemical Genomics Center for the curve fitting and curve classification that is available for open access (www.ncgc.nih.gov) [15]. The results from experiments with control compounds were analyzed with Prism^® ^software (GraphPad, San Diego, CA) for the curve fitting and EC_50_/IC_50_ calculation.

## RESULTS AND DISCUSSION

**Fig. (1):**
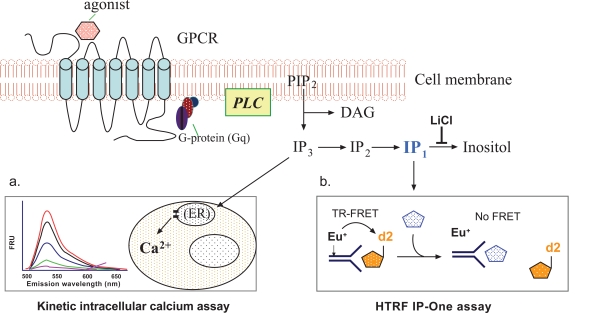
Schematic representation of the principles for the intracellular calcium assay and the IP-One assay.

Agonist binding to a Gq-coupled GPCR results in activation of PLC, which hydrolyzes phosphatidylinositol bisphosphate (PIP_2_) to form two second messengers, IP_3_ and DAG (Fig. **1**). While DAG activates protein kinase C (PKC), IP_3_ activates the IP_3_ receptor on the endoplasmic reticulum (ER) that results in an efflux of Ca^2+^ from ER to cytoplasm and elevation of intracellular free Ca^2+^. These events transfer the extracellular signal to biological responses in cells [10]. The IP_3_ is very rapidly hydrolyzed to IP_2_, then to IP_1_ and finally to inositol by a series of enzymes; meanwhile Ca^2+^ is quickly pumped back into ER by a calcium pump on the ER membrane. Both processes effectively switch off the IP_3_ and Ca^2+^ signaling. The time frames for intracellular free Ca^2+^ and IP_3_ measurements are usually within a few seconds to a few minutes [3,17]. For compound screens, the changes in intracellular free Ca^2+^ concentration can be measured by fluorescence calcium dyes with a fluorescence kinetic plate reader (Fig. **1a**). As an alternative method for assessing the function of Gq-coupled GPCRs, IP_3_ or total IPs including IP_1_, IP_2_ and IP_3_ are usually measured in the presence of lithium, an inhibitor of inositol monophosphatase (Fig. **1b**). In this newly available HTRF based IP-One assay, an IP_1_ specific antibody is used to detect the labeled IP_1_ tracer. When the IP_1 _level in the cell lysate elevates that is proportional to an increase in IP_3_, the TR-FRET between the labeled antibody and IP_1_ tracer is disrupted (Fig. **1b**).

### IP-One Assay in the 1536-Well Plate Format

The original IP-One assay was developed in 96/384-well plate formats and requires a medium change for adherent cells or use of freshly detached suspension cells [14,18]. The additional cell wash step is obviously not suitable for HTS especially in 1536-well plate format. The automated robotic operation in HTS requires continuous supply of live cells over many hours. Adherent cells cultured overnight in assay plates are usually the convenient source of live cells for this purpose. Alternatively, for screenings using the compound pre-dispensed assay plates, suspension cells are needed in which the cells can be washed in batch before dispensing into the assay plates. Thus, we had miniaturized the IP-One assay in both adherent and suspension cell modes in 1536-well plates.

### Plate Wash *vs* no Plate Wash

**Fig. (2):**
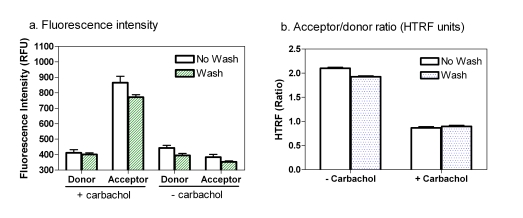
Comparison between the “wash” and “no wash” protocols for the IP-One assay. (**a**) The fluorescence intensity of the fluorescence donor and acceptor when assayed with the “wash” or “no wash” protocol. (**b**) HTRF ratios determined with the “wash” or “no wash” protocol.

Medium removing or cell wash was suggested previously for the IP-One assay in order to reduce to potential interference from serum and phosphates present in the cell culture medium, reducing the liquid volume in wells, and maintaining the final lithium concentration [14,18]. We performed an assay with a cell wash step (cell culture medium was changed to buffer) to compare with that without a cell wash using adherent cells cultured overnight in 1536-well plates. LiCl, an inositol monophosphatase inhibitor, was added at a final concentration of 50 mM to accumulate inositol phosphates including IP_1_. We found that eliminating cell wash step did not reduce the fluorescence intensities of either donor or acceptor fluorophores or change the acceptor/donor fluorescence ratio (Fig. **2**). In fact, the signal-to-basal (S/B) ratio was slightly higher in the assay without a cell wash compared with that with a cell wash. This indicates that the cell wash step in 1536-well plates with small assay volume is not necessary for this IP-One assay. The IP-One assay in homogenous assay format is more suitable for HTS. The small amount (4 μl/well) of cell culture media in 1536-well plates might reduce the negative effect of phosphate containing medium on the IP-One assay.

### Adherent Cells *vs*. Suspension Cells

**Fig. (3):**
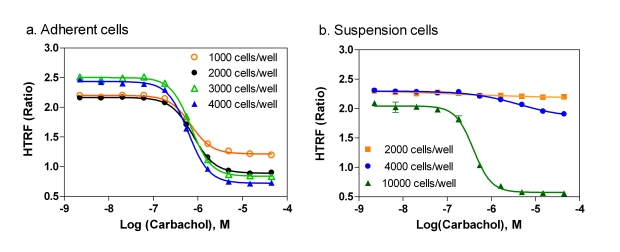
Comparison between the adherent cell mode and the suspension cell mode for the IP-One assay. (**a**) Carbachol dose-response curves for the IP-One assay were determined with different densities of adherent cells. (**b**) Carbachol dose-response curves for the IP-One assay were determined with different densities of suspension cells.

In the adherent cell mode of the IP-One assay, resuspended frozen cells were used for cell plating in 1536-well assay plates instead of freshly cultured cells because frozen cells were readily available and convenient for HTS [19]. We found that the S/B ratios in the adherent cell mode with 1000, 2000, 3000, 4000 cells/well were 1.84, 2.40, 3.01 and 3.39 fold, respectively. The EC_50_ values of Carbachol remained the same in these cell densities (Fig. **3a**). In the suspension cell mode of IP-One assay, freshly detached cultured cells were resuspended in a phosphates-free buffer included in the IP-One assay kit. The IP-One assay was then performed immediately after the cells were dispensed to 1536-well assay plates. We found that 2000 and 4000 cells/well in the suspension cell mode did not produce the same levels of signal as these in the adherent cell mode (Fig. **3b**). A 3.56 fold of S/B ratio was obtained in the suspension assay mode only after the cell density increased to 10,000 cells/well as it was reported previously in 384-well plates [14,18]. The results indicate that a higher cell density in the suspension cell mode is needed and the adherent cell mode requires much less cells. Frozen cells were tested in the suspension cell mode and they did not produce enough signals, while use of cell culture medium with 10% FBS produced similar signal as the phosphate-free buffer in the suspension cell mode (data not shown). Two reasons are likely to account for the difference of cell numbers in these two assay modes. First, cell numbers increase or even double after overnight culture in the adherent cell mode. Second, the cell health condition in assay plates can also affect the assay signal. The process for preparing freshly suspended cells involves cell detachment, centrifugation, resuspension and plating that can cause the stress and/or injury to cells. Thus, more cell numbers are needed to produce the same amount of IP_1_ in the suspension cell mode because the freshly plated cells are not fully recovered from the stress/injury, whereas the cells in the adherent cell mode are recovered after overnight cell culture and have better responses in the experiment. We selected 2000 cells/well for the compound screen in the adherent cell mode for the rest of study considering the cells reached 80 to 90 % confluence after overnight incubation. A density of 8000 to 10,000 cells/well in 1536-well plates is recommended for the IP-One assay in the suspension cell mode.

The results also indicated that the EC_50_ values of carbachol obtained from both adherent and suspension cells were similar (Fig. **3**). This result was consistent with previous reports that the responses of frozen cells after overnight culture were comparable to the freshly detached cells in several other assays [19,20].

**Fig. (4):**
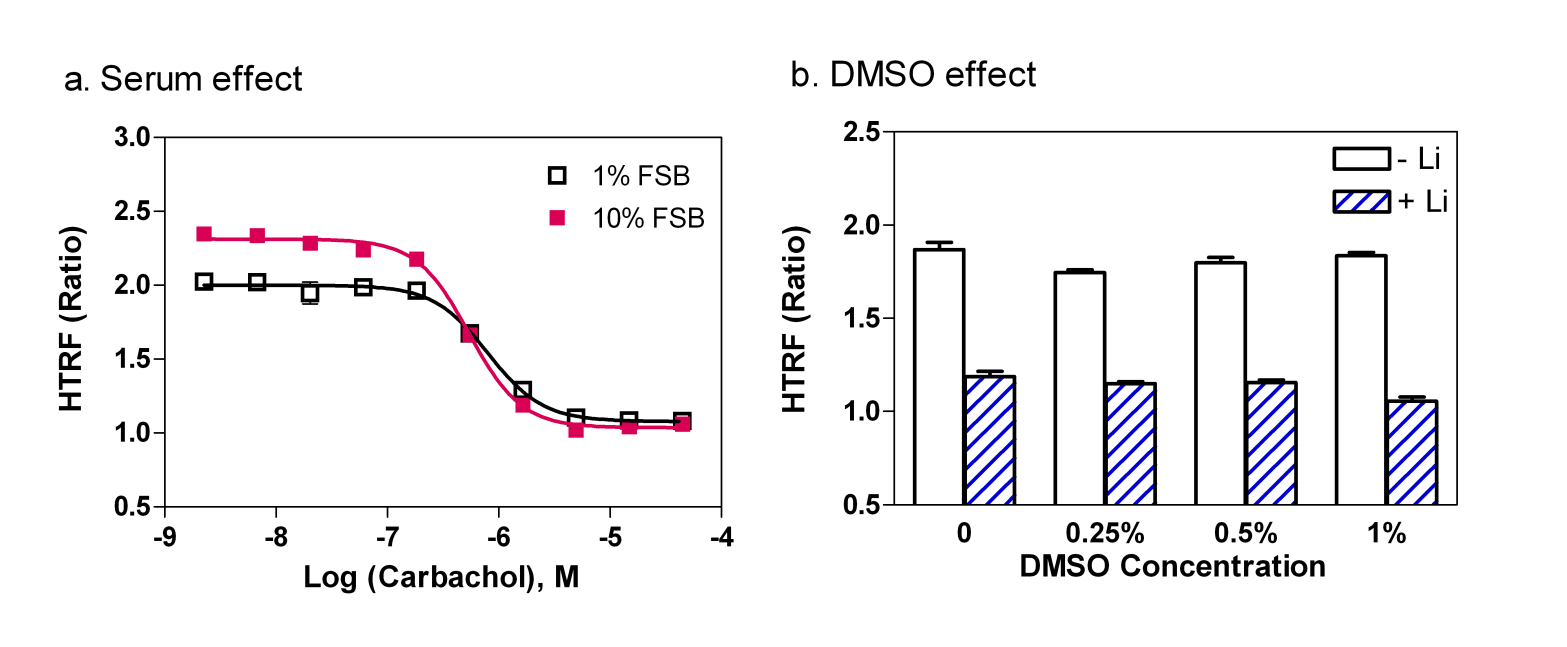
Effects of serum and DMSO on the performance of the IP-One assay. (**a**) Effect of FBS concentration on the assay performance. (**b**) Effect of DMSO concentration on the assay performance.

The effect of FBS concentration on the assay performance was also tested in the M_1_-CHO cells. We found that although the EC_50 _value of Carbachol was not changed in the 1% FBS medium compared to that in the 10 % FBS medium (Fig. **4a**), the S/B ratio decreased from 2.21 fold in 10 % FBS to 1.87 fold in 1 % FBS medium. This can be explained by the decreased amount of IP_1_ produced in 1% FBS cultured cells compared with the 10% FBS cultured cells. With a consideration of better S/B ratio for the antagonist screening, 10 % FBS medium was selected for this IP-One assay. We also found that the DMSO concentration was tolerated up to 1 % in this IP-One assay (Fig. **4b**). If the use of 10% FBS concentration is a concern, either 1% FBS medium or the suspension cell mode with a higher cell density can be used.

### Comparison of Agonist Responses Between the IP-One and Intracellular Calcium Assays

**Fig. (5):**
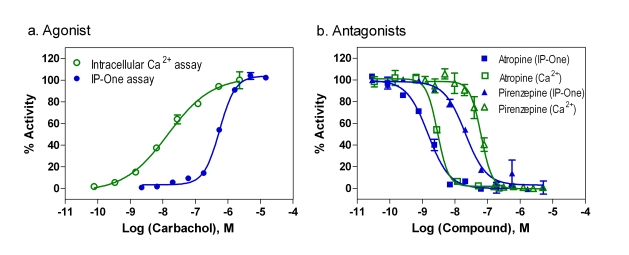
Comparison of agonist and antagonist activities determined in IP-One and intracellular calcium assays. (**a**) Concentration responses of carbachol. (**b**) Concentration-responses of muscarinic acetylcholine antagonists. The EC80 amount of agonist, carbachol, was used in both assays (5 μM for the IP-One assay and 0.1 μM for the intracellular calcium assay).

Carbachol concentration responses in the same M_1_-CHO cells were determined in both the IP-One assay and intracellular calcium assay with a fluorescent calcium dye using a fluorescence kinetic plate reader. We found that EC_50_ value of carbachol was 535 nM determined in the IP-One assay (Fig. **5a**), similar to that reported previously obtained in the radioactive IP_1_ assay with ^3^H-inositol incorporation [11,14]. The EC_50_ value of carbachol determined from the intracellular calcium assay in the fluorescence kinetic measurement was 15.1 nM that is similar to that of 23 nM reported elsewhere (www.invitrogen.com/) but was 35 fold more potent than that determined from the IP-One assay (Fig. **5a**). This discrepancy of carbachol potency can be explained by the receptor reserve (spare receptor) theory. The carbachol dissociation constant for the receptor binding was reported previously to be 1-3 μM [21,22] while its EC_50_ for the intracellular calcium assay was 15.1 to 23 nM. This suggested that only a small portion of receptors in this M_1_-CHO cell line need to be activated to stimulate a full calcium response. This discrepancy of the carbachol potency might be partly due to the different agonist incubation time in two assays. The agonist response in the intracellular calcium assay was measured at 1-2 minutes as it reaches its peak whereas the agonist incubation time in the IP-One assay was 30 minutes in order to reach its peak response. In addition, other unknown factors related to a specific receptor may be responsible for this discrepancy.

The agonist responses of three other Gq-coupled GPCR cell lines including the free fatty acid receptor 1 (FFAR1), the vasopressin V1b receptor and the neuropeptide S (NPS) receptor were also determined in both IP-One and intracellular calcium assays. We found that the EC_50_ values of agonists determined in the intracellular calcium assay for FFAR1 receptor and NPS receptor were similar to these determined from IP-One assay (Table **1**). The EC_50_ values of NPS in the intracellular calcium assay and IP-One assay were 0.22 and 0.41 nM, respectively. The Kd value of NPS receptor binding was 0.33 nM reported previously [23], correlating well with the activities determined from both functional assays. However, the agonist activity to V1b receptor was 34.4 times more potent than that obtained in the IP-One assay (Table **1**). The extent of left shift of the agonist responses in the intracellular calcium assay with the V1b receptors was similar to that observed in the M_1_-CHO cell line.

### Comparison of Antagonist Responses Between the IP-One and Intracellular Calcium Assays

Two muscarinic acetylcholine receptor antagonists, atropine and pirenzepine were tested in both assays to compare their activities. Atropine is a nonselective muscarinic acetylcholine receptor antagonist and pirenzepine is a M_1_ selective antagonist. In the experiments with antagonist screening, an EC_80_ amount of carbachol (agonist) was added to stimulate the M_1_ receptor responses. Because of the difference of agonist potency in these two assays, 0.1 and 5 μM Carbachol were used in the intracellular calcium assay and IP-One assay, respectively. Under these conditions, the S/B ratios were similar in both the IP-One assay and intracellular calcium assay for measurement of antagonist activities. The IC_50_ value of atropine was 2.93 nM determined from the intracellular calcium assay which is similar to 1.82 nM obtained from the IP-One assay (Fig. **5b**). For pirenzepine, the IC_50_ values were 63.3 and 20.8 nM, respectively in intracellular calcium assay and IP-One assay (Fig. **5b**). This result indicates that the potencies of antagonists are similar and comparable in both assays using EC_80_ amount of agonist.

### LOPAC Library Screenings in the IP-One and Intracellular Calcium Assays

**Fig. (6):**
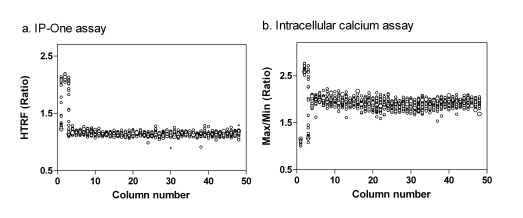
DMSO plate tests in a M_1_-CHO cell line for the intracellular calcium and IP-One assays. (**a**) Scatter plot of the results from IP-One assay. All wells were stimulated the EC_80_ amount of carbachol (4 μM). Column 2 was received 50 μM atropine (IC_100_). (**b**) Scatter plot of the results from intracellular calcium assay. All wells in this plate were stimulated with 0.1 μM carbachol (in EC_80_). Column 1 was treated with 50 μM atropine (IC_100_) and column 2 was added with the EC_100_ amount of carbachol.

A DMSO plate was first tested to evaluate both assays in the antagonist screening mode with the EC_80_ concentration of carbachol. The IP-One assay in 1536-well plates was performed in a homogenous assay format (Table **2**). The S/B ratio was 1.86 fold and Z’ factor was 0.78 (Fig. **6a**), indicating a robust screening assay. The no-wash calcium dye was used in the intracellular calcium assay with a newly available kinetic plate reader specifically designed for 1536-well plate assays (Table **3**). The S/B ratio and Z’ determined from the intracellular calcium assay were 1.80 fold and 0.64, respectively (Fig. **6b**). Although the S/B ratios in both assays were smaller than these in the agonist assay mode, the Z’ factors were above 0.5 indicating that both assay were robust for compound screening.

The results of both the IP-One assay and intracellular calcium assays are calculated ratiometrically which compensates for the relatively small S/B ratios for the assay performance. Both IP-One and intracellular calcium assays described here do not require cell wash nor medium aspiration and are thus suitable for high throughput screening. While the intracellular calcium assay requires a costly kinetic fluorescence plate reader, the IP-One assay can be carried out using a standard fluorescence plate reader that is obviously a convenient and cost-effective alternative for compound screening with Gq-coupled GPCRs.

We then screened the LOPAC library in a quantitative high throughput screening (qHTS) format with both the IP-One and the intracellular calcium assays. Since all compounds were titrated in 15-concentrations, the active compounds were evaluated by their potencies and the curve classes as it was discussed in detail previously [15]. The results of cholinergic antagonists were particularly examined in details and their potencies in two assays are compared in Table **4**.

### Correlation of Antagonist Activities Between IP-One and Intracellular Calcium Assays

**Fig. (7):**
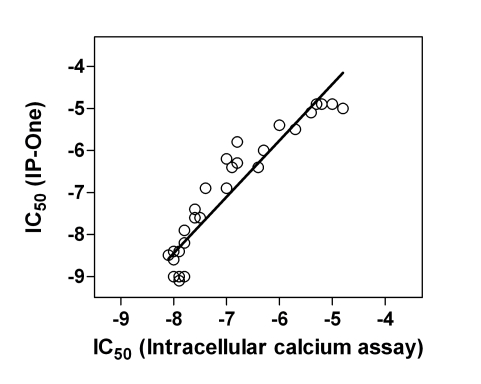
Correlation of known cholinergic antagonist activities between the IP-One and intracellular calcium assays. The LOPAC collection was screened in the qHTS format with both the IP-One assay and the calcium assay. The IC_50_ values of 30 know cholinergic antagonists determined from both screens were correlated.

The LOPAC collection contains 30 known cholinergic antagonists including M1 specific, other subtype specific and nonselective antagonists. The IC_50_ values of these 30 known antagonists determined from the IP-One assay correlated with these measured from the intracellular calcium assay (Fig. **7**). Together with the results of atropine and pirenzepine, it further demonstrated that the antagonist activities determined in this IP-One assay were similar to those obtained from the intracellular calcium assay in the kinetic assay mode.

Taken together, the intracellular calcium assay measures the real-time kinetic changes of free Ca^2+^ in live cells. It is a reliable assay method for screening compounds acting on Gq-coupled GPCRs; however, this method requires a special kinetic plate reader with the liquid handling capability for the addition of agonist to all the wells in an assay plate simultaneously. The tips on the multiple channel pipette head must be washed extensively before being used for the next assay plate. This tip washing step significantly limits the screening throughput and assay plate density in the previous version of instruments including FDSS-6000 and FLIPR Tetra. We have applied a newly designed FDSS-7000 plate reader which has one 1536-well pipetting head and three wash stations with sonicators. It can effectively and quickly wash tips for reuse and make the intracellular calcium assays robust in a 1536-well plate format; although the instrument is still costly.

Alternatively, the IP-One assay can be carried out with a standard plate reader without the requirement of a kinetic measurement. The HTRF assay format of the IP-One assay also reduces the well-to-well variation because the result is calculated in a ratiometric manner which can minimize the variations caused by cell number and dispensing errors among wells. The IP-One assay is also a homogenous assay format and has been miniaturized to 1536-well plates. The EC_50_ values of the agonists determined in the IP-One assay were similar to these reported in the radioactive IP assay. Thus, it is a useful alternative method for compound screening with Gq-protein coupled GPCRs. Compared with the intracellular calcium assay, the EC_50_ values of agonists in the IP-One assay could be less potent but the antagonist potencies are similar in both assays.

In summary, we have optimized and miniaturized an IP-One assay for the M_1_R, V1bR, FFAR1, and NPSR cell lines. The potencies of agonists determined from this IP-One assay are similar to these obtained from the traditional radioactive IP_3_ assay. The EC_50_ values of agonists determined from some GPCRs (FFAR1 and NPSR) were similar in both intracellular calcium and IP-One assays, while these determined from others (M_1_R and V1bR) were less potent in the IP-One assay, probably due to the receptor reserve. In comparison with the intracellular calcium assay using the kinetic detection mode, the potencies of antagonist responses are similar in both assays. The IP-One assay is homogenous and robust without requirement of a costly kinetic plate reader. These results indicate that the TR-FRET based IP-One assay is an alternative method to screen large sizes of compound collections for the Gq-coupled GPCRs.

## Figures and Tables

**Table 1.** EC50 Values of four Gq-Coupled GPCRs Determined in the IP-One and Intracellular Calcium Assays

GPCR	Agonist	EC_50_(nM) (calcium assay) (a)	EC_50_(nM) (IP-One assay) (b)	Ratio b/a
M_1_R	Carbachol	16.1	535	33.2
V1bR	Vasopressin	0.032	1.10	34.4
FFAR1	GW9508	5700	6300	1.1
NPSR	NPS	0.22	0.41	1.9

**Table 2.** IP-One Assay Protocol in 1536-Well Plate Format

Step	Parameter	Value	Description
1	Cells	4 μL	2000 cells/well
2	Incubation	24 hrs	37°C, 5% CO_2_
3	Compound	23 nl	in DMSO dilution
4	Incubation	10 min	37°C, 5% CO_2_
4	Reagent	1 μl	Carbachol (EC_80_)
5	Incubation	30 min	37°C, 5% CO_2_
7	Reagents	1 μl each	IP1-d2 and Eu-anti-IP1-Ab in cell lysis buffer, dispensing separately
8	Incubation	30 min	Room temperature
9	Detection	HTRF mode	ViewLux plate reader

**Table 3.** Calcium Assay Protocol in 1536-Well Plate Format

Step	Parameter	Value	Description
1	Cells	3 μl	2000 cells/well
2	Incubation	16-20 hrs	37°C, 5% CO_2_
3	Reagent	3 μl	No-wash calcium dye
4	Incubation	60 min	37°C, 5% CO_2_
5	Compound	23 nl	in DMSO dilution
6	Incubation	10 min	Room temperature
7	Detection	Basal signal	10 reads at 1 Hz
8	Reagent	2 μl	Carbachol (EC_80_)
9	Detection	Antagonist response	4-minute reading at 1Hz

**Table 4.** IC50 Values (μM) of 30 Known Cholinergic Antagonists Determined in the IP-One and Intracellular Calcium Assays in M1-CHO Cells

Compound	Property	Calcium Assay	IP-One Assay
Tropicamide	MAR anta. (M4)	2.00	3.16
Atropine methyl bromide	MAR anta.	0.01	0.003
Atropine sulfate	MAR anta.	0.01	0.001
Atropine methyl nitrate	MAR anta.	0.013	0.001
BW 284c51	Ach. Inh.	6.31	12.6
Aminobenztropine	MAR anta.	0.016	0.003
Benztropine mesylate	MAR anta.	0.008	0.013
4-DAMP methiodide	M_3_ and M_5_ MAR anta.	0.016	0.006
Dicyclomine hydrochloride	MAR anta.	0.039	0.631
4-DAMP mustard hydrochloride	Irreversible MAR anta.	0.10	0.126
L-Hyoscyamine	MAR anta.	0.01	0.501
Ipratropium bromide	MAR anta.	0.016	0.004
DL-Homatropine hydrobromide	MAR anta.	0.16	0.001
Hexahydro-sila-difenidol hydrochloride, p-fluoro analog	MAR anta.	0.16	1.58
MG 624	NAR anta.	0.13	0.398
Methoctramine tetrahydrochloride	MAR anta.(M2 selective)	1.00	3.98
Orphenadrine hydrochloride	MAR anta.	0.50	1.00
Pancuronium bromide	skeletal muscle relaxant	10.0	12.6
Oxybutynin Chloride	MAR anta.	0.025	0.039
Pirenzepine dihydrochloride	MAR anta.	0.10	0.126
Propantheline bromide	MAR anta.	0.012	0.001
(-)-Scopolamine hydrobromide	MAR anta.	0.012	0.004
(-)-Scopolamine methyl nitrate	MAR anta.	0.012	0.001
(-)-Scopolamine,n-Butyl-, bromide	MAR anta.	0.16	0.50
(-)-Scopolamine methyl bromide	MAR anta.	0.013	0.001
Trihexyphenidyl hydrochloride	MAR anta.	0.025	0.0251
N,N,N-trimethyl-1-(4-trans-stilbenoxy)-2-propylammonium	NAR anta.	3.98	7.94
Telenzepine dihydrochloride	MAR anta. (M1 select)	0.032	0.025
WB 64	MAR anta.	0.398	0.398
9-Amino-1,2,3,4-tetrahydroacridine	Ach inh.	15.8	10.0

Note: MAR - muscarinic acetylcholine receptor; anta. – antagonist; Ach. Inh. – acetylcholinesterase inhibitor; NAR - Nicotinic acetylcholine receptor.
